# Behavioral Evidence for Local Reduction of Aphid-Induced Resistance

**DOI:** 10.1673/031.007.4801

**Published:** 2007-09-11

**Authors:** Ernesto Prado, W. Fred Tjallingii

**Affiliations:** ^1^ INIA, La Platina, Casilla 439-3, Santiago Chile; ^2^ WUR Laboratory of Entomology, POB 3081, 6700 EH, Wageningen, The Netherlands

**Keywords:** systemic resistance, insect-plant interactions, pepper, wheat, broccoli, electrical penetration graph

## Abstract

Twenty-five aphids of three different species, *Brevicoryne brassicae* L, *Myzus persicae* Schulzer, and *Rhopalosiphum padi* L(Hemiptera: Aphididae) were each allowed to infest leaves of a young plant of their respective host plant species for 4 days, except that the oldest expanded leaf (the ‘systemic’ leaf) was kept free of aphids. Each preinfested plant thus had two types of leaves, local leaves (preinfested with aphids) and one systemic leaf, the oldest true leaf that had been kept free of aphids.In subsequent choice tests, settling preference of aphids was tested between the systemic leaves of these preinfested plants and leaves of uninfested control plants. Aphids significantly preferred leaves of control plants in settling choice tests, thus indicating some resistance in the systemic (uninfested) leaves of the preinfested plants. Plant penetration and feeding was further investigated with the electrical penetration graph (EPG) technique using *B. brassicae* on broccoli, its host plant. The tests included both the systemic and infested (local) leaves of preinfested plants as well as control plants. Aphid-induced resistance in systemic leaves was confirmed by EPG data. Fewer aphids showed phloem feeding on systemic leaves, only 30% as compared to 100% on control leaves. However, on local leaves 100% of the aphids showed phloem feeding, indicating a strong reduction in systemic resistance induced by aphids in these leaves. Phloem factors are the main cause of induced resistance. The possible roles of different phases of salivary secretion in systemically-induced resistance and its local reduction are discussed. In addition to these preinfestation experiments, EPG tests were also done on aphids on broccoli plants that were exposed to volatiles emitted from aphid-infested broccoli plants to compare probing behavior of volatile-induced resistance with systemic resistance due to preinfestation. Phloem factors also appeared to be involved in volatile-induced resistance, although some behavioral details differed.

## Introduction

Plant attack by insect herbivores has been shown to increase plant defenses, generally referred to as induced plant resistance (Karban and Baldwin, 1997). On the other hand, the opposite effect, induced plant susceptibility, has been reported as well (Prado and Tjallingii 1997). Plant responses induced by signals from the attacking insects have been described, including transcriptional changes (Moran and Thompson, 2001; Klinger et al. 2005; De Vos et al. 2005), and changed metabolic cascades effected by the signalling hormone, jasmonic acid (Thaler 2002). In the latter case, the produced metabolic products can be volatile, and their release may affect the attacking organisms as well as their natural enemies in the ecosystem (Chamberlain et al. 2001). In addition to insect-to-plant, and plant-to-insect signals, plant-to-plant signals have also been reported, i.e. signals released by attacked plants that appear to induce resistance to insects in uninfested neighboring plants (Pettersson et al. 1996; Dicke and Bruin 2001).

Many piercing-sucking herbivores, particularly phloem feeders such as aphids, scales, and whiteflies, have apterous or sessile stages that complete their whole life span, or an important part of it, on the same plant and often their subsequent offspring generations do so as well. As a result, these insects have to rely on the infested plant for a long time. This raises the question of how these colonizing insects deal with the resistance they induce. This study was done to determine whether plant acceptance behavior by an aphid changed after induction of resistance in a host plant by the same aphid species. Three aphid-plant combinations were used and induced effects were observed in a simple choice test to detect settling preference. Since we are primarily interested in effects on probing behavior, we used the electrical penetration graph (EPG) technique (Tjallingii 1988) in one aphid-plant combination. Behavioral analysis using EPG waveforms allows localizing the induced effects to specific plant tissues. A similar EPG study has recently been done by Dugravot et al. 2007 with *Myzus persicae* on potato plants.

**Table 1.**
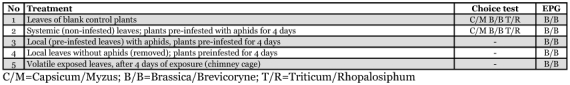
Treatments of leaves of plant/aphid combinations used in the two experiments, choice tests and EPG recordings.

## Materials and Methods

### Plants and aphids

Plants and aphid colonies were reared in the greenhouse at 16:8 L:D and 22 ± 3°C. The aphids had originally been collected in the field (Santiago de Chile). Three plant-aphid combinations were used and plants were at the following stages at the time of infestation, and aphids had the following morph, age and rearing origin, respectively, 1) Sweet pepper, *Capsicum annuum* L. cv. “Resistant” was used as potted plants at a stage of two true leaves with *Myzus persicae* Schulzer (combination C/M), apterous virginoparous adults, about 4 days after the last molt, from a colony reared on *C. annuum.* 2) Broccoli, *Brassica oleracea* L, cv. “Arcadia” was used as potted plants at a stage of two true leaves with *Brevicoryne brassicae* L. (combination B/B), apterous virginoparous adults, about 4 days after the last molt, from a colony reared on *B. oleracea*. 3) Wheat, *Triticum aestivum* L. cv. Chagual was used as potted plants at a stage of four true leaves with *Rhopalosiphum padi* (L) (combination T/R), apterous virginoparous adults, about 4 days after the last molt, from a colony reared on *T. aestivum*.

### Preinfestation and dual choice settling tests

The host plants of each aphid species, in the stage mentioned above, were infested within a cage with 25 aphids. The first true leaf that was fully expanded was kept free of aphids with non-woven, anti-aphid tissue that was tightened around the petiole. Each preinfested plant thus had two types of leaves, local leaves (preinfested with aphids) and one systemic leaf, the oldest true leaf that had been kept free of aphids. Subsequent choice tests started 4 days after this preinfestation. Systemic leaves of preinfested plants (Table 1, treatment 2) were tested against blank leaves of control plants (Table 1, treatment 1) in a settling choice test. Test plant pots were laid horizontally on a table and one leaf of each plant was inserted under the lid of a 10 cm Petri dish on filter paper under day light and photo period conditions. Each Petri dish thus included a choice situation between two leaves, a treated and a control leaf for 10 aphids that were released under the lid. Settling of the aphids on each leaf was scored after 5, 24 and 48h, at 22 ± 3°C. The number of choice test replicates used within plant/aphid combinations C/M, B/B and T/R were 23, 25, and 35, respectively. Aphids that were not on the leaves during scoring were excluded, and score results were expressed as percentages of aphids on any of the leaves.

**Figure 1.**
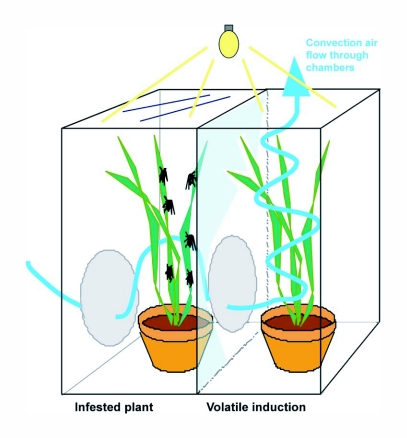
Chimney set-up to induce resistance by plant volatiles produced by plants infested with aphids. Left compartment completely closed but with a convention airflow trough with two (grey circular) screened parts. Right compartment with open top: the ‘chimney’ part.

### EPG recording

The electrical penetration graph (EPG) technique (DC system, Tjallingii, 1988) was used to compare probing (plant penetration and feeding) of *B. brassicae* on broccoli leaves with the 5 different treatments. Aphids were collected and individuals were gently brushed to remove some cuticular wax from their dorsum. A small droplet of water based silver glue was then applied to the dorsum to attach a gold wire electrode of 20 µm diameter and about 2 cm long. A 4-channel amplifier (model Giga-4, Wageningen University) allowed simultaneous recording of 4 aphids during 8 hours. Data acquisition (Keithley's DAS800, 12 bit ISA plug-in card) at 100 Hz conversion rate was used to store the signals on a PC hard disk. EPG recording and waveform analysis was mediated by DOS software (Stylet 3.7, Wageningen University). For each of the 5 treatments 20 replicate aphids were EPG recorded.

**Figure 2.**
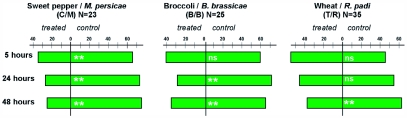
Settling choice test results. Settling preference of treated leaves (Table 1, treatment 2) vs. control leaves scored after 5, 24, and 48 hours. Although avoidance of treated leaves was clear in all three plant/aphid combinations, the wheat/ *Rhopalosiphum padi* combination showed this avoidance only after 48h. Bars represent 100%, i.e. all insects on any leaf in each replicate, (25–35 choice tests in total for each plant/aphid combination) were statistically tested with Mann-Whitney U-test (p < 0.005)

### EPGs from aphid induced plants

Broccoli plants were preinfested by *B. brassicae* as in the choice test setup. After 4 days, 8 hour EPGs were recorded from aphids on blank leaves of control plants (Table 1, treatment 1), on uninfested systemic leaves (treatment 2), on preinfested local leaves with aphids (treatment 3) and without aphids (treatment 4), i.e. after the preinfesting aphids had been removed.

### EPGs from volatile exposed plants

EPGs were also recorded on leaves of broccoli plants that had been exposed to volatiles from *B. brassicae* infested broccoli plants, using “chimney cages” (Petterson *et al*., 1996). Plants were exposed to volatiles of infested plants for 4 days in these chimney cages (Figure 2). After the volatile exposure EPGs were recorded for 8 hours on these plants (treatment 5) (Table 1).

### Statistics

Choice test results were tested by Mann-Whitney's U-test. In EPG results, mean numbers and total durations of waveforms were tested by Kruskal-Wallis, followed by pair-wise comparison, treatments vs. control only, whereas Fischer”s 2×2 test (treatments vs. control) was used for percentages of aphids showing a certain waveform. Significance levels of p<0.05 and p<0.01 are indicated.

## Results and Discussion

### Settling choice

Aphids in choice tests preferred settling on blank control leaves to systemic leaves in all three plant-aphid combinations. Avoidance of systemic leaves appeared sooner in *Capsicum*/*M. persicae* combination than in the broccoli/*B*, *brassicae* combination, whereas and *R. padi* on wheat showed avoidance only after 48 hours (Figure 2). Settling avoidance suggests systemic spread of the aphid-induced resistance, although the induced resistance in the uninfested leaf might have been caused also by volatiles from the preinfested plant parts. If systemic, it remains unclear whether this effect is only due to signals acting via sieve tubes, xylem vessels, or by communication via other pathways as well. The fully expanded leaf in which systemic effects occurred can be considered as the source leaf, physiologically, mainly with a basipetal phloem transport. So, if signaling from the local leaves occurred downward in the phloem, some upward xylem signaling might have played a role subsequently. As humidity might have increased in the Petri dish arena, the xylem signaling presumably stopped after some time in the leaves tested. Although the 48-hour Petri dish exposure might have influenced signaling, and other physiological features, such effects occurred in both treated and control leaves.

### EPG results

The results are summarized by the relative time (%) aphids spent probing/non-probing (complementary figures) and the relative numbers of aphids (%) showing phloem activities on leaves with different treatments (Table 2). On systemic leaves, and leaves of plants exposed to volatiles, the aphids exhibited reduced probing time and fewer aphids showed phloem activities, as compared to blank controls. This suggests that in these treatments some resistance to aphids was induced. This has been reported earlier in plants exposed to volatiles (Pettersson et al. 1996). Nevertheless, our results indicate some differences between resistance due to volatile exposure and direct aphid attack. In leaves exposed to volatiles the reduced total time of sieve element salivation (E1) was not statistically significant and the percentage of aphids with (E2) and sustained phloem ingestion (sap feeding; sE2) was less reduced than in systemic leaves of directly attacked plants. The most important result was that there was almost no resistance shown in local leaves of preinfested plants, with or without aphids (Table 2, treatments 3 and 4).

**Table 2.**
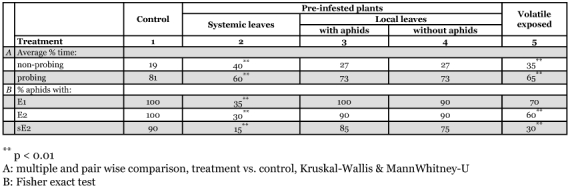
General stylet penetration features. Relative figures (%) on main EPG results. Fractions of time probing and non-probing are taken from the first probe to the end of recording. Fractions (%) of all aphids (N=20), i.e. showing phloem activities, E2, sieve element salivation; E2 sieve element ingestion; sE2 sustained sieve element ingestion (>10 min). Treatments 2 and 5 are showing aphid-induced resistance, in treatments 3 and 4 the induced resistance appears to be absent, locally suppressed as we suggest. Treatments in accordance to Table 1.

**Table 3.**
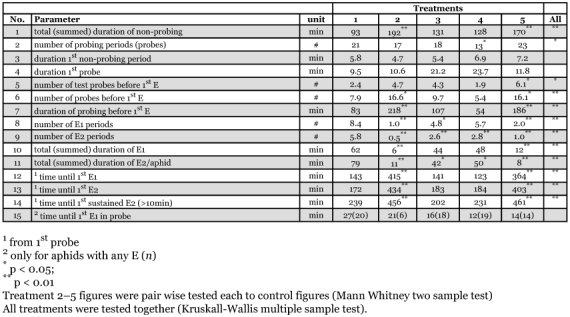
Detailed features of stylet penetration from EPGs. E1 and E2 refer to EPG waveforms, reflecting salivation into and ingestion from a sieve element, respectively. For treatment numbers, see Table 1.

More details of induced resistance were derived from further analysis of the EPG results. In agreement with the foregoing relative non-probing time (Table 2), the total duration of non-probing (shown in minutes, Table 3, parameter 1) increased in systemic as well as in volatile-induced leaves, although the number of probes (Table 3, parameter 2) remained the same in these treatments as compared to control leaves. Thus, non-probing was longer but probes did not occur more frequently. The duration of the first non-probing period (Table 3, parameter 3), during which surface factors are encountered by aphids (Alvarez et al., 2006), did not differ between treated and control leaves. Several early probing activities, indicated as the stylet pathway phase, are important, if not essential, for establishing plant suitability by aphids. The pathway phase represents intercellular stylet penetration from epidermis to phloem, during which aphids also puncture nearly every cell encountered. Typically, these intracellular punctures only last for about 5 seconds, and then stylets are withdrawn to continue the intercellular stylet path (Tjallingii and Hogen Esch 1993). Sap samples taken during these intracellular punctures (Martin et al. 1997) are likely to contain chemical cues influencing stylet penetration and host plant selection behavior of aphids (Montllor, 1999). No differences were shown in EPGs between treatments with respect to some often used parameters such as the duration of the first probe and the number of short test probes (i.e probes < 3 min.) before the first phloem phase (Table 3, parameters 4 and 5). This indicates that no resistance factors were detected by the aphids in the epidermis and shallow mesophyll (Gabrys et al. 1997), at least not in preinfested plants. In contrast, in plants exposed to volatiles, the number of test probes before any phloem activity (parameter 5) did increase, suggesting at least some volatiles induced resistance in these tissue layers.

**Figure 3.**
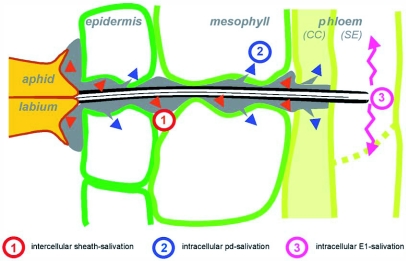
Three identified salivary secretion phases into the plant during stylet penetration by aphids. (1) Gelling saliva (red arrows), forming the salivary sheath, (2) watery saliva (blue arrows), intracellularly secreted during brief stylet punctures (pd waveform), (3) watery saliva (purple arrows), into phloem sieve elements (E1 waveform), preceding phloem feeding. Salivation (3) might be mainly responsible for the induced resistance that is systemically spread, whereas salivation (1) and (2) may have more local effects.

The total number of probes before the first phloem phase, and their total duration (parameters 6 and 7), increased in aphids on systemic leaves and on plants exposed to volatiles (treatments 2 and 5). These probes include those that were longer than 3 minutes and deeper into tissues. During the probes before phloem activities were seen, the stylets presumably reached the phloem as such but aphids did not switch to phloem activities in treatments 2 and 5. Aphids generally do perform many brief punctures of phloem companion cells and sieve elements without switching to phloem activities (Tjallingii and Hogen Esch 1993). The reduced total number and total time of both phloem activities, phloem salivation (E1) and phloem ingestion (E2) in treatments 2 and 5 (parameters 8 to 11) do support this idea. Also, this is supported by delayed first phloem salivation (E1), phloem ingestion (E2), and sustained phloem ingestion (sE2; parameters 12 to 14). Within probes with phloem activity, the preceding duration of stylet pathway (parameter 15) was not significantly different between aphids on control and treated leaves (though seeming longer than on local leaves). Resistance reduced the number of probes with phloem activities but very likely not phloem contacts, brief punctures of companion cells and sieve elements and, therefore, the time until phloem activities (parameter 12–14). Finally, the fact that the aphids on systemic and volatile-exposed leaves showed first phloem activities within a probe no later than on control leaves, suggests that they might habituate to phloem resistance factors during the earlier probes. Thus the resistance factor(s) is not located before, but more likely inside, the phloem. Again, resistance effects were clearly shown in aphids on systemic leaves and those on plants exposed to volatiles (treatments 2 and 5), but not in aphids on the preinfested local leaves (treatments 3 and 4). It should be mentioned here that total phloem feeding (parameter 11) is also significantly shorter in aphids on the local leaves, suggesting that although resistance is reduced in aphids on these leaves, feeding still is not as good as on blank control leaves. In our opinion, this demonstrates that each step toward sustained phloem feeding in aphids needs a stimulus to start as well to be maintained.

The EPG results support the choice test results of aphid-induced systemic resistance due to preinfestation and, additionally, they indicate that the resistance factor(s) are phloem located. Moreover, the EPG data clearly show that aphid-induced resistance is reduced in local preinfested leaves, protecting the aphid colony against its own induced resistance. We have not investigated spatial and temporal aspects of this reduction, nor for how long aphid-induced systemic resistance remained active. How inter-specific the induced resistance and its reduction acts, i.e. against other aphid species or in mixed infestations also needs to be studied further. Dugravot et al, (2006) recently confirmed systemic resistance and local reduction in potato infested by the aphid *M. persicae*, regardless whether preinfestation was conspecific or heterospecific by *Macrosiphum euphorbiae*; inverse effects were not studied. Many questions remain with respect to the nature of signals and their pathways, transcription changes, metabolites produced, and the final mode of action to the aphids whether sensory (Wensler and Filshie, 1969) and/or physical-chemically (Knoblauch and Van Bel, 1998). Nevertheless, our primary question, how aphids deal with their own induced resistance, is answered in the sense that aphids appear to be able to reduce significantly, and possibly suppress this resistance locally. This reduction is not due to individual habituation in the aphids, but it is an aphid-induced plant property. This holds at least for all three plant-aphid combinations we investigated.

The resistance induced by exposure to volatiles from aphid-infested plants seems to be very similar to resistance induced by preinfestation. However, there are a few remarkable differences. First, the number of test probes shown before the first phloem activity (Table 3, parameter 3) is higher. This also suggests that some non-phloem factors play a role in volatile-induced resistance. These non-phloem tissues are closer to the leaf surface and have been in direct contact to the volatiles entering via the stomata. In addition, resistance due to volatile exposure showed weaker phloem effects (% of aphids with E1, E2 and sE2, Table 2). Therefore, this may plead against a pure volatile-mediated cause of preinfestation induced resistance, and makes a systemic signal route more likely.

Finally, we would like to speculate about how the observed long range and local effects might have been caused. It is likely that the aphid's saliva secreted into plants plays a crucial role. This saliva may include a number of different signals for the plant. There is experimental evidence for three periods of salivary secretion into plants (Figure 3; Tjallingii and Cherqui 1999; Tjallingii 2006). The first period comprises sheath salivation within the stylet pathway. Sheath saliva is gelling saliva that envelops the stylet bundle in their intercellular position (Figure 3, saliva 1). Also within the pathway small amounts of watery (non-gelling) saliva is secreted, injected into cells during the characteristic short intracellular punctures (pd waveforms; saliva 2 in Figure 3) (Martin et al., 1997). Furthermore there is watery salivation during both sieve element activities (waveforms E1 and E2). However, the E2 salivate does not reach the plant because the pressure of the phloem sap forces this saliva into the food canal during phloem ingestion (Prado and Tjallingii 1994). We think that sieve element salivation (saliva 3, Figure 3) may be mainly responsible for the long-range systemic effects because it is injected directly into the plant's transport system. This saliva is thought to have also a function in the suppression of primary wound responses in the sieve elements (Knoblauch and Van Bel, 1998; Will and Van Bel, 2006; Will et al, 2007), The other two salivary secretion phases during formation of the stylet pathway (Figure 3, saliva 1 and 2) may possibly cause the short-range local reduction of induced resistance as it is not secreted in the vascular system, Phloem effects in general may primarily reflect metabolic activities or changes in the phloem companion cells so that the difference between the short and long -range effects presumably comes from different signals, short-range from the mesophyll and long range from the sieve elements. Saliva may not be the signal itself. After transduction in companion cells it can be any plant signal, but that is rather speculative as well.

## Abbreviations

EPG -: electrical penetration graph
